# The Engrafting of Nerves

**Published:** 1845-07

**Authors:** 


*The Engrafting of Nerves.*—M. Flourens, in reference to some experiments made by M. Tavignot, proving the possibility of engrafting nerves one on the other, reminded the Academy that he had published, some years since, similar experiments, with like results. He had seen the interlaced reunion of several nerves; for instance, the superior nerves with the inferior of the brachial plexus, and even the cervical nerves with the pneumogastric. In all these cases there was complete reunion, and in some, a complete return of function. (*See* "Memoirs of the Academy," vol. xiii. p. 14, and his Experimental Researches into the Functions of the Nervous System," &c., p. 272, *et seq.*—*London Lancet.*

